# Commentary: Understanding the public voices and researchers speaking into the 5G narrative

**DOI:** 10.3389/fpubh.2024.1461515

**Published:** 2024-08-01

**Authors:** Frank de Vocht, Patricia Albers

**Affiliations:** Population Health Sciences, Bristol Medical School, University of Bristol, Bristol, United Kingdom

**Keywords:** bias (epidemiology), conflict of interest (COI), 5G, mobile phones, cellphones, radiofrequency (RF), white hat bias

## 1 Introduction

Weller and McCredden published work in this journal to provide information around the voices speaking publicly about 5G telecommunications and potential adverse health effects (1). Their work is, at least in part, motivated by our work on how the initial narrative around 5G was shaped (2), but misrepresents, or possibly misunderstands, various aspects of our work. We take the opportunity here to address these.

## 2 Misunderstandings and misrepresentations

Weller and McCredden in their article claim we categorized “reputable industry-independent scientists who speak against 5G” under “activism” or labeled them as “white hats.” This is an important misunderstanding of our work, and which subsequently results in misrepresentations of our work in their manuscript throughout. We outline in our Methodology that we *a priori* classified each publication in our study as “Articles were assigned as “industry” or “activism” depending on whether the articles report links between the authors and either industry or campaigning organizations related to 5G in particular or mobile phones more broadly, or as “independent” otherwise,” and “In case no such links were reported, a basic internet search was performed to identify unreported link.” There is no value judgment in this classification, but Weller and McCredden erroneously claim this classification is a judgment of individuals and their scientific credibility. They go as far as stating that authors were somehow “demoted,” and, inappropriately, make reference to “discredit the scientist” strategies previously used by the tobacco industry. Such subjective judgments on individuals are extremely susceptible to the “appeal to authority” fallacy, and were not part of our work.

Instead, we focused on the research methodologies of the included peer-reviewed publications. For each publication we discussed the publication type, whether detail of the methodology for evidence synthesis was included, and whether a publication included an assessment of the scientific quality of the studies it referenced. In a second step, we then compared these between publications classified as associated to activism, to industry, or as independent. We observed that works associated to activism more often employed less rigorous methods with a higher potential for biased conclusions. In relation to industry-funded research, such research practices, including non-systematic inclusion of references and lack of explicit assessment of study quality, have been described as indications of industry bias (3). However, in the case of our research such practices were more often observed in publications with links to activism, which is indicative of “white hat bias.” White hat bias has been described as bias leading to distortion of information in the service of what may be perceived to be righteous ends (4), and, like its mirror image “industry bias,” refers to a piece or body of work and not to an individual's expertise or authority. As such, individuals are not “white hat biaser,” as Weller and McCredden misconstrue, any more than they are “publication biasers” or “information biasers.”

Weller and McCredden further state that (financial) links to industry can be the only conflicts of interest. In fact, they explicitly state that “researchers are affiliated with professional advocacy organizations, which should not be misconstrued as a conflict of interest, as suggested by de Vocht and Albers (2), but instead, regarded as part of their ethical obligations.” This is at best a naïve interpretation, but in any case at variance with the contemporary interpretation of what construes CoIs which also include (in addition to industry and other financial CoIs) a variety of non-financial CoIs including those originating from particular concerns, ideals, and predilections (5). Indeed, this journal does explicitly include such in their COI Rules (https://www.frontiersin.org/guidelines/policies-and-publication-ethics#conflicts-of-interest). Ironically, Weller and McCredden's statement that affiliation to an advocacy organization cannot be a CoI but is an ethical obligation, is an indication of aforementioned “white hat bias.”

A third misrepresentation of our work by Weller and McCredden is their statement that we claim that “public concerns have been described in a recent opinion piece (2) as representing only a small “pocket” of society whose opinions originate from beliefs in conspiracy theories or mere “perceptions” of health risks.” We state that “5G has also been met with resistance from anti-5G campaigning organizations supported by pockets of the general public,” and while we make reference to the perceptions of increased radiofrequency (RF) radiation exposure and to the influence of 5G-related conspiracy elsewhere, it is disappointing Weller and McCredden choose to explicitly link these as if they were our words. They erroneously paraphrase us throughout and claim that “... mixed results and conclusions not supporting increased risks” in our manuscript is the opinion of the authors. We do not make any statement on whether 5G is causally related to adverse health effects. In fact, the full quote from our publication “With the increasing contribution from independent and industry-linked authors over the covered time period, the narrative shifts from the exclusive reporting of increased risks of all biological or health effects covered to predominantly descriptions of mixed results and conclusions not supporting increased risks,” is a clear description of how the narrative around 5G and health effects changed over the 2018–2021 time period covered by our work. Indeed, we explicitly state that “… there is no clear answer (yet) whether the resulting narrative from the peer-reviewed literature describes an overestimation of risks as a result of articles with links to campaigning organizations, or whether later contributions from authors with links to industry, and possibly most independent authors, at the latter stages of the critical window describe an underestimation of true causal associations, or whether their combined evaluation will inform future evidence synthesis closer to “the truth”.”

## 3 Discussion

We believe the analysis of research methodology, in particular in evidence synthesis, to be an important contributor to public health information and policy, as is the evaluation of the voices who make up the debates. However, accuracy in such analyses is important, and herewith we hope we have clarified the many errors in Weller and McCredden's work with respect to our analyses.

## Author contributions

FV: Conceptualization, Writing – original draft, Writing – review & editing. PA: Conceptualization, Writing – original draft, Writing – review & editing.
